# Modelling human CNS injury with human neural stem cells in 2- and 3-Dimensional cultures

**DOI:** 10.1038/s41598-020-62906-y

**Published:** 2020-04-22

**Authors:** Barbora Vagaska, Olivia Gillham, Patrizia Ferretti

**Affiliations:** Stem Cells and Regenerative Medicine Section, UCL Great Ormond Street Institute of Child Health, University College London, London, WC1N 1EH UK

**Keywords:** Biological models, Neural stem cells

## Abstract

The adult human central nervous system (CNS) has very limited regenerative capability, and injury at the cellular and molecular level cannot be studied *in vivo*. Modelling neural damage in human systems is crucial to identifying species-specific responses to injury and potentially neurotoxic compounds leading to development of more effective neuroprotective agents. Hence we developed human neural stem cell (hNSC) 3-dimensional (3D) cultures and tested their potential for modelling neural insults, including hypoxic-ischaemic and Ca^2+^-dependent injury. Standard 3D conditions for rodent cells support neuroblastoma lines used as human CNS models, but not hNSCs, but in all cases changes in culture architecture alter gene expression. Importantly, response to damage differs in 2D and 3D cultures and this is not due to reduced drug accessibility. Together, this study highlights the impact of culture cytoarchitecture on hNSC phenotype and damage response, indicating that 3D models may be better predictors of *in vivo* response to damage and compound toxicity.

## Introduction

In light of the practical and ethical limitations of human research, animal models provide fundamental insight into the central nervous system (CNS) development, injury, disease, as well as the possibility of studying and screening putative therapeutic compounds. However, there is an increasing appreciation of the limitations of animal research, which often fails to reliably predict successful human clinical trials. Key examples of this are hypoxia-ischemia either in newborns (1–8 cases per 1000 births in the Western world) or adults (cerebral stroke) and traumatic injuries to the CNS^1–3^. While animal models have significantly furthered understanding of the mechanisms of injury and repair, and various neuroprotective agents have proven effective in animal injury models, this has not translated into effective treatments in humans^4–6^. Such failures to translate animal studies to successful human clinical trials are often attributed to problems with methodological and study design. However, species-specific differences in drug processing, genetic interactions and molecular mechanisms of action can be argued to be fundamental to therapeutic translation. Further studies on the cell ular mechanisms of human CNS injury and repair are therefore needed in order to develop better therapeutic strategies and bridge the gap between animal models and human clinical trials^7,8^.

Three dimensional (3D) culture models have been rapidly emerging as a means to study human cells *in vitro*. While still in many respects rather challenging, because more complex to maintain and analyse, they reduce the strain and artificial responses which cells must undergo in order to adapt to the flat, stiff surfaces of 2D monolayer culture. A 3D architecture provides a more tissue-like environment in which cell-cell and cell-matrix interact in all dimensions and cells have more biologically relevant exposure to diffusible factors^9,10^. Whereas the use of iPSCs (induced pluripotent stem cells) derived from patients to study neural diseases has been rapidly expanding, there is still a lack of human injury models particularly in 3D^11–13^.

Here we have used human neural stem cells (hNSCs) derived from embryonic and foetal brain as a cell source for setting up 3D cultures and injury models^10,14,15^. The advantage of hNSCs over iPSCs or hESCs (human embryonic stem cells) for this purpose is that they can proliferate extensively *in vitro*, have the potential to differentiate towards neurons, astrocytes and oligodendrocytes and are less demanding than iPSCs to maintain in culture^14–18^. Furthermore, in comparison with iPSCs, hNSCs differentiation protocols are in general much shorter, as they do not require the initial phase of inducing the cells towards neural progenitors.

We set out to establish and evaluate a 3D human cell culture system using hydrogel scaffolds in order to mimic *in vivo* CNS tissue architecture while maintaining reproducibility and minimising variation found in self-organised 3D tissue models such as neurospheres (neural cell aggregates grown in suspension), or organoids. While very useful for modelling development and certain diseases, organoids do not yet provide the reproducibility required to be used for pharmacological screening and are expensive to produce^19–21^. We first assessed the behaviour of different human neural cells, neuroblastoma, hNSCs and hNSC-derived neurons, in different hydrogels and polystyrene scaffolds in 3D as compared to 2D monolayer cultures. This led to selecting a 3D system consisting of Matrigel and Collagen I for modelling neural damage induced by calcium imbalance and hypoxic-ischemic injury. Hypoxic injury and reperfusion was modelled by reducing oxygen levels and removing glucose form the medium (oxygen-glucose deprivation, OGD) for different lengths of time prior to returning the cells to normal conditions. In order to simulate the intracellular responses which occur following traumatic injury and ultimately result in apoptosis, thapsigargin, a sarco/endoplasmic reticulum calcium ATPase (SERCA) inhibitor, was used to induce intracellular Ca^2+^ release in hNSCs and neurons. Here we show that hNSCs respond differently to both types of injury when grown in 3D cultures, as compared to 2D. Further, hNSC-derived neurons were found to be more resistant to calcium dependent injury than hNSCs.

## Results

### Human neural stem cells and neuroblastoma cell behaviour in 3D cultures

**Figure 1:**
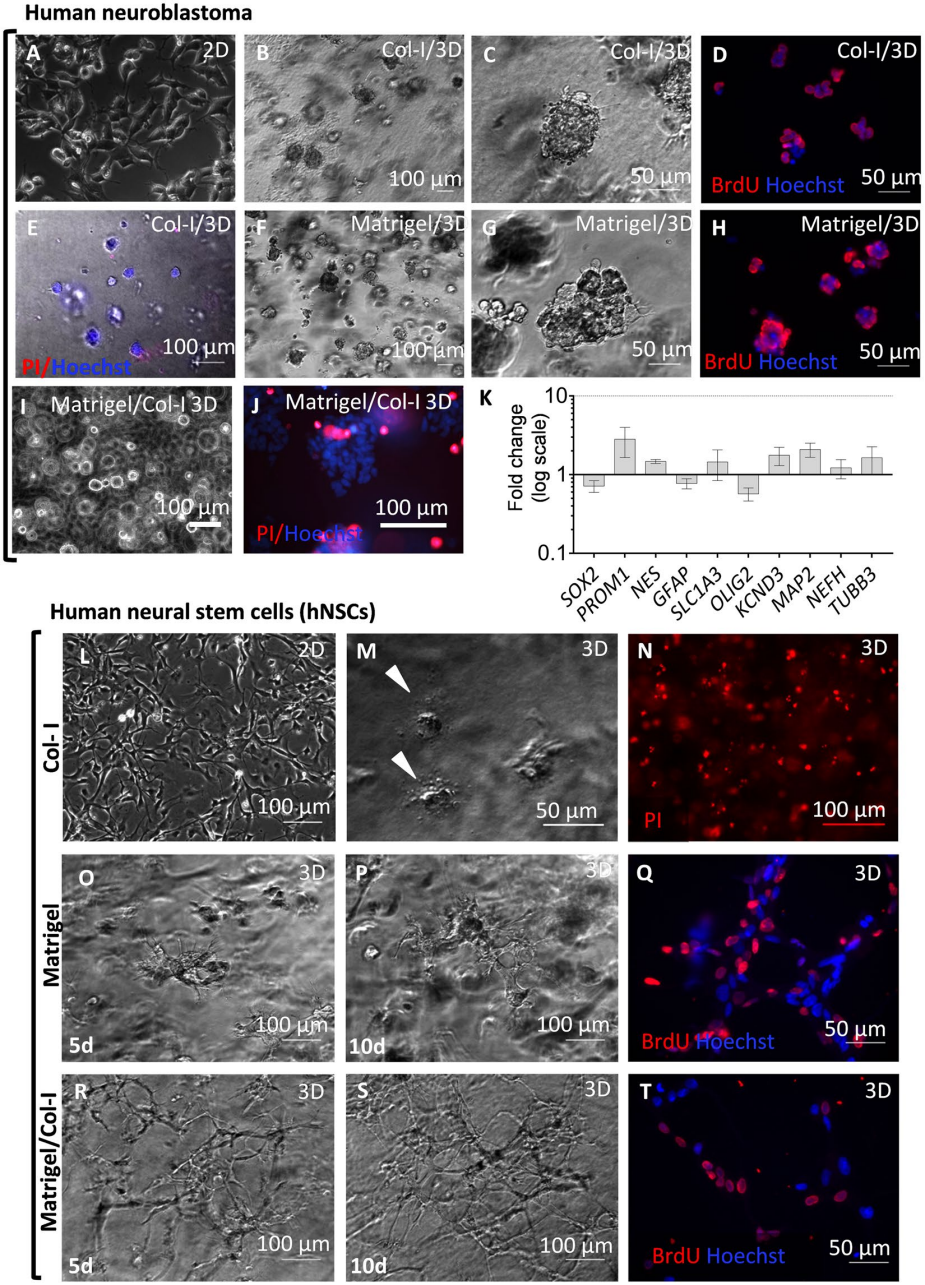
Behaviour of neuroblastoma cells and human neural stem cells (hNSCs) in different 3 dimensional (3D) extracellular matrices (collagen gel and Matrigel) assessed by live imaging, cell death/survival and gene expression analysis. (**A–J**) Neuroblastoma (LAN-5) cells 5 days after seeding: **(A)** LAN-5 cell monolayers on plastic; (**B–E)** LAN-5 cells grown in collagen I (Col-I) 3D cultures; note formation of tumor-like aggregates, extensive proliferation, as indicated by BrdU incorporation (red), and very limited cell death, as indicated by propidium iodide (PI, red) labelling; nuclei (blue) are detected by Hoechst 33258 dye staining; **(F–H)** LAN-5 cells grown in Matrigel-based 3D cultures display similar morphology and proliferation to those cultured in Col-I hydrogels. (**I**,**J)** LAN-5 cells grown in Matrigel/Col-I 3D cultures; limited cell death is observed. (**K)** RT-qPCR of LAN-5 cells grown in 2D and 3D Matrigel/Col-I hydrogels for 5 days (n = 3 biological replicates); note differences in expression of neural stem cells markers. NTC: no template control. (**L–T** cultures: **(J)** hNSC monolayers grown in the presence of laminin; (**L–N**) hNSCs after 2 days in Col-I hydrogel; note extensive cell death, as indicated by cell morphology and propidium iodide (PI) staining. **(O–Q)** hNSCs grown in Matrigel-based 3D cultures; formation of cell networks is already observed at 5 days (5d) and shows increased complexity at 10 days (10d); BrdU incorporation is indicative of proliferative activity. **(R–T)** hNSCs behave in a similar fashion when grown in a mixed hydrogel (Matrigel/Col-I, 2.25/1.35 mg/ml) rather than in Matrigel alone; note extensive BrDU labelling.

To develop a 3D culture model of human neural cells we first compared behaviour of neuroblastoma cell lines that can be easily and reproducibly expanded and neuronally differentiated with primary cultures of human neural stem cells (hNSCs) in two different 3D hydrogels, collagen type I (Col-I) gel and Matrigel (Fig. 1 and Supplementary Fig. 1). Single-cell suspensions were embedded in Col- I or Matrigel and cell behaviour monitored at different times. In standard 2D cultures, the LAN-5 cell line displayed typical morphological features of N-type neuroblastoma cells, with small cell bodies, little cytoplasm and short neurites (Fig. 1A). In 3D cultures, most neuroblastoma cells initially were round shaped with thin neurites extending into the matrix, but by two days in culture they displayed a tendency to aggregate rather than spreading out (Supplementary Fig. 1A). These aggregates were observed within the whole thickness of the hydrogel; their size increased over time, becoming tighter and with a tumour-like appearance by day 10 in culture (Fig. 1B,C). Proliferative activity and viability of the neuroblastoma cells embedded in collagen hydrogels was confirmed by BrdU and propidium iodide (PI) staining respectively (Fig. 1D,E). Comparable behaviour was observed when LAN-5 were cultured in Matrigel or in Matrigel/Col-I hydrogels (Fig. 1F–J). Rapid cell aggregation in 3D cultures was observed also in two other neuroblastoma cell lines, SH-SY5Y and IMR-32, that like LAN-5 readily undergo neuronal differentiation and are widely used as neuronal models (Supplementary Fig. 1B,C). We also investigated whether the morphological changes observed  were paralleled by changes in gene expression. By 5 days in 3D cultures, neuroblastoma cells in collagen gels showed up-regulation of neuronal markers and *PROM1* (CD133, prominin) and slight down-regulation of glial markers (e.g. *OLIG2* and *GFAP)* in 3D as compared to 2D (Fig. 1I).

As Col-I hydrogel supported survival, growth and neuronal differentiation of neuroblastoma cells (Fig. 1; Supplementary Fig. 1) and rodent cells^22–24^, we tested whether this was the case also for hNSCs, that in 2D cultures depend on laminin for attachment and spreading (Fig. 1L). In Col-I hydrogels, hNSCs failed to extend processes into the matrix and 5 days after seeding most cells had died, as indicated by PI staining, with cell debris visible throughout the gel (Fig. 1L–N). In contrast, in 100% Matrigel, hNSCs were viable and started to spread within 24 hours of culture (Fig. 1O–Q). They extended thin filopodia into the matrix and, by 5 days, had established connections with neighbouring cells forming network-like structures. hNSCs were also actively proliferating as indicated by the increased cell density observed microscopically in live cells and BrdU incorporation (Fig. 1Q). We then assessed behaviour of hNSCs in hydrogels containing different proportions of Matrigel and Col-I. hNSCs were viable in mixed gels at all tested concentrations indicated in the Methods (Supplementary Fig. 2; Fig. 1R–T). As shown in Fig. 1R–T, in Matrigel/Col-I gels (3.4 mg/ml/1 mg/ml), hNSCs showed a similar behaviour to that observed in 100% Matrigel; however, their shape was more elongated than in Matrigel alone, with more rapid formation of a network structure that had already achieved higher complexity by 5 days in culture. Extensive BrdU incorporation was observed in these cultures (Fig. 1T).

**Figure 2:**
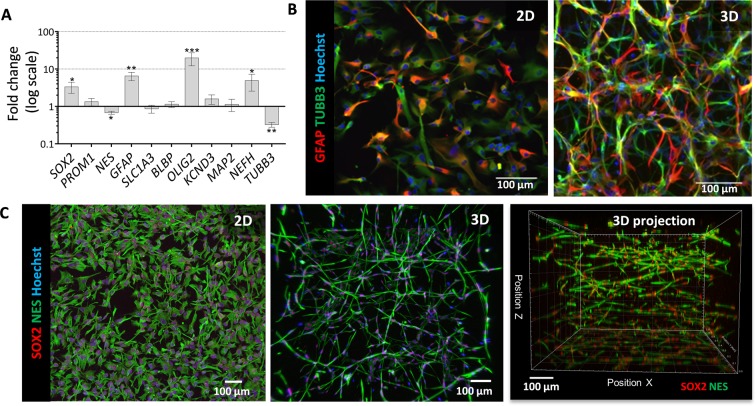
Assessment of hNSCs behaviour cultured and differentiated in 2D and 3D hydrogel cultures by RT-qPCR and immunostaining. (**A**) Expression of neural marker transcripts of hNSCs in 3D versus 2D 5 days after seeding. Graph represents mean expression of three independent hNSC lines, each carried out with biological quadruplets. *P < 0.05. (**B**) ß3-TUBULIN (TUBB3) and GFAP in hNSCs in 2D and 3D hydrogel. (**C**) Expression of NESTIN (NES) and SOX2 in hNSCs in 2D and 3D hydrogel (Matrigel/Col-I, 2.25/1.35 mg/ml) together with XZ/YZ projection and 3D volume projection generated from the confocal images; note changes in the morphology and organisation of hNSCs when cultured in 3D. Nuclei (blue) are detected by Hoechst dye staining.

Analysis of neural marker transcripts by RT-qPCR in 3 hNSC lines showed significant up-regulation of *SOX2*, *GFAP*, *OLIG2* and *NEFH* (encoding NF200) mRNAs, and down-regulation of *ß3-TUBULIN*, and to a lesser extent *NES* (encoding NESTIN) mRNAs in 3D cultures as compared to 2D 5 days after seeding (Fig. 2A). We further investigated hNSCs in 3D culture and their potential to undergo neuronal differentiation by immunofluorescence and confocal microscopy. Consistent with the gene expression data, ß3-TUBULIN and GFAP proteins were detected in both 2D and 3D hNSCs (Fig. 2B,C). Immunostaining for these cytoskeletal proteins in 3D revealed the complex network formed by the hNSCs within the gel at 7 days of culture.

Neuronal differentiation of hNSCs takes several weeks, and hydrogels were unstable and showed some degradation over the time required for hNSCs neuronal differentiation. In addition, we found that passaging already differentiated neurones resulted in extensive cell loss. In order to explore strategies for circumventing technical issues, we initially tested different protocols using neuroblastoma cells, SH-SY5Y, that, upon retinoic acid (RA) induction, undergo neuronal differentiation over days rather than weeks. As neuroblastoma cells in 3D rapidly aggregated, we tested the possibility of pre-differentiating them along the neuronal lineage in 2D and then seeding them in a mixed Matrigel/Col-I hydrogel for further differentiation and maturation in 3D. Following seeding in 3D, SH-SY5Y cells quickly recovered their morphology and neurite branching was observed by 2 days. At this stage, the medium was supplemented with the neurotrophic factor, BDNF; SH-SY5Y processes further elongated forming intricate neurite architecture (Supplementary Fig. 3A). The differentiated cultures strongly expressed neuronal markers (NF200, β3-TUBULIN, MAP2) and the neuroblast marker doublecortin (DCX) was detected in some cells (Supplementary Fig. 3B). Moreover, staining with Sv2 antibody revealed the presence of synaptic vesicles along the processes suggestive of extensive vesicular transport. Up-regulation of genes associated with neuronal differentiation (*MAP2*, choline acetyl transferase (*ChAT*) and tyrosine hydroxylase (*TH*)) was detected also by RT-qPCR (Supplementary Fig. 3C). Whereas up-regulation of *MAP2* and *ChAT* transcripts was comparable in 2D and 3D cultures, a larger TH fold increase was observed in 3D than in 2D, possibly indicating that the 3D environment is more suitable for dopaminergic differentiation.

**Figure 3:**
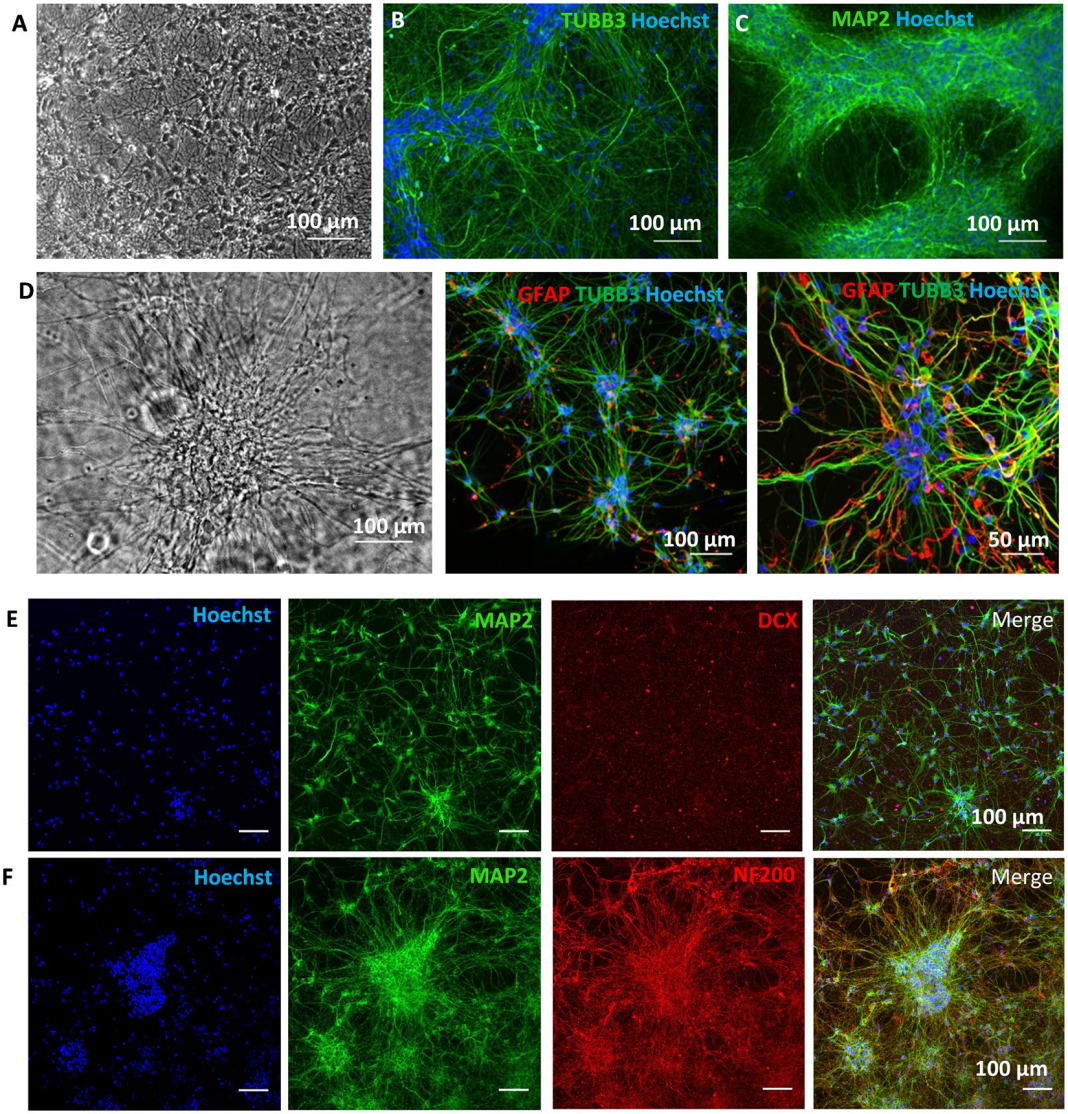
Assessment of hNSCs differentiated in 2D and 3D hydrogel cultures by immunostaining. (**A–C**) Phase contrast image and immunostaining for the neuronal markers, TUBB3 and MAP2, in hNSCs in 2D cultures after 4 weeks of neuronal differentiation. (**D**) Phase contrast image and double-staining for ß3-TUBULIN (TUBB3) and GFAP in 3D culture after 2 weeks of differentiation in 2D and 2 weeks differentiation in 3D culture. Note the mixed cell phenotypes, with many cells with processes strongly stained for ß3-TUBULIN, others expressing the stem/glial marker GFAP, and a few double-labelled cells indicative of differentiation still in progress. (**E)** Double-staining for MAP2 and DCX in 3D neural cultures differentiated as in (**D**). (**F)** Double-staining for MAP2 and NF200 in 3D culture differentiated as in (**D**). Nuclei (blue) are detected by Hoechst 33258 dye staining.

We then used the same strategy for inducing neuronal differentiation of hNSCs taking into consideration the longer differentiation time required by these cells. hNSCs were pre-differentiated in 2D cultures for 2 weeks prior to enzymatic detachment and then either maintained in 2D or seeded in mixed Matrigel/Col-I hydrogels for further maturation for at least another 2 weeks (Fig. 3). Once in 3D, the differentiating cells reacquired their neuronal morphology within a couple of days; they had small cell bodies that tended to cluster together and thin processes that made contact with the neighbouring cell body clusters, whereas hNSCs differentiated in 2D were more spread out (Fig. 3A–D). Immunocytochemical staining of neuronally-induced hNSCs for β3-TUBULIN, MAP2 and NF200 revealed fine, long and extensively branching processes (Fig. 3D–F). The presence of a different smaller population of cells was highlighted by GFAP staining, suggesting the presence of glial cells at this stage of differentiation, but staining for the neuroblast marker, doublecortin (DCX) was largely negative (Fig. 2D,F).

**Figure 4:**
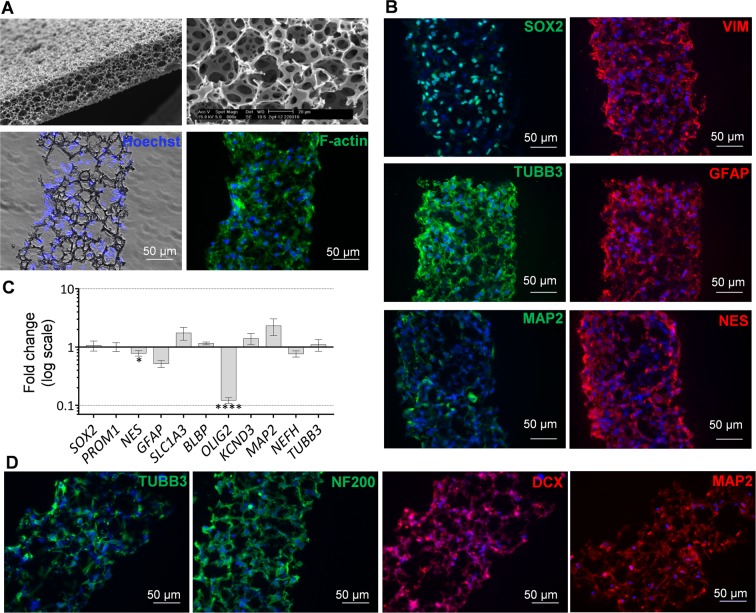
Analysis of hNSCs cultured and differentiated in 3D polystyrene scaffolds (Alvetex) by RT-qPCR and immunostaining. (**A**) Scaffolds have a highly porous structure as shown by scanning electron micrography (top panels taken from http://reinnervate.com); by 8 days after seeding onto laminin pre-coated scaffolds, hNSCs have colonized the scaffold throughout its full thickness as indicated by the nuclear (Hoechst 33258, blue) and cytoskeletal (F-actin, green) staining (bottom panels). (**B)** Representative expression of hNSCs markers in sections of scaffolds including SOX2, vimentin (VIM), ß3-TUBULIN (TUBB3), GFAP and NESTIN (NES) assessed by immunocytochemistry. Nuclei are counterstained in blue with Hoechst 33258. (**C)** RT-qPCR analysis of neural markers in one hNSC line cultured in 3D scaffolds for 5 days (n = 3 biological replicates); changes in transcript levels in 3D cultures in comparison with 2D controls are observed. (**D)** Immunocytochemical analysis of scaffold sections with hNSCs differentiated towards the neuronal lineage for 5 weeks; note expression of neuronal marker such as TUBB3, doublecortin (DCX), neurofilaments (NF200) and MAP2.

Longer-term differentiation of hNSCs in 3D cultures based on hydrogels is technically challenging because of the low stability of the matrices in long-term cultures as cells reorganize and contract the substrate. Therefore, we also evaluated hNSCs grown in a synthetic 3D scaffold, Alvetex, which is stable over time. This scaffold is made of tissue culture grade polystyrene, is 200 μm thick and has more than 90% porosity with pores of different sizes and good connectivity as shown in the scanning electron micrographs provided by the manufacturer (Fig. 4A). After coating the scaffold with laminin, hNSCs were able to attach, grow and colonize the full thickness of the scaffold within 8 days in culture, as demonstrated in scaffold sections (Fig. 4A). As shown by immunocytochemistry, hNSCs continued to express the neural cell markers detected in 2D, such as SOX2, vimentin, NESTIN, GFAP, ß3-TUBULIN, as well as low levels of MAP2 (Fig. 4B). Comparison of gene expression in 2D and in the Alvetex scaffold by RT-qPCR in one cell line showed much smaller changes than in the hydrogel, apart from *OLIG2* which was greatly reduced in the 3D cultures, and *NESTIN* that showed a small but significant reduction (Fig. 4C). The ability of hNSCs to undergo neuronal differentiation in Alvetex was also assessed. Immunocytochemical analysis after 5 weeks of neuronal differentiation confirmed neuronal morphology with long processes and strong expression of neuronal markers such as β3-TUBULIN, MAP2, and neurofilaments, but doublecortin expression was still detectable (Fig. 3D).

While Alvetex provided a viable 3D environment for hNSC growth and neuronal differentiation, it is a rigid porous polystyrene scaffold which limits cell self-organization and is not amenable to *in vivo* cell monitoring. Additionally, polystyrene and hydrogel scaffolds were found to differentially impact upon hNSC gene expression. Finally, diffusion of compounds through a biological matrix and its mechanical properties more closely mimic brain tissue, and so we favoured hydrogel-based cultures for developing human injury models.

### Modelling human neural injury in 2D and 3D cultures

In order to quantify possible differences in hNSC behaviour in 2D versus 3D following injury, rapid and reliable quantitative analysis of cell numbers and viability is needed. Following comparison and optimization of two luminescence-based cell viability assays (based either on reduction of the MT substrate in live cells or on ATP measurement following cell lysis), the assay measuring luminescent signal depending on ATP levels showed lower variability in our system (Supplementary Fig. 4). Hence it was selected as the semi-quantitative assay for assessing whether the 3D cultures could be utilized to study the effect of oxygen-glucose deprivation (OGD), a simple model widely used to mimic aspects of cerebral stroke^25^ and of calcium imbalance, which occurs after traumatic injury, using thapsigargin as previously reported in 2D cultures^8^.

**Figure 5:**
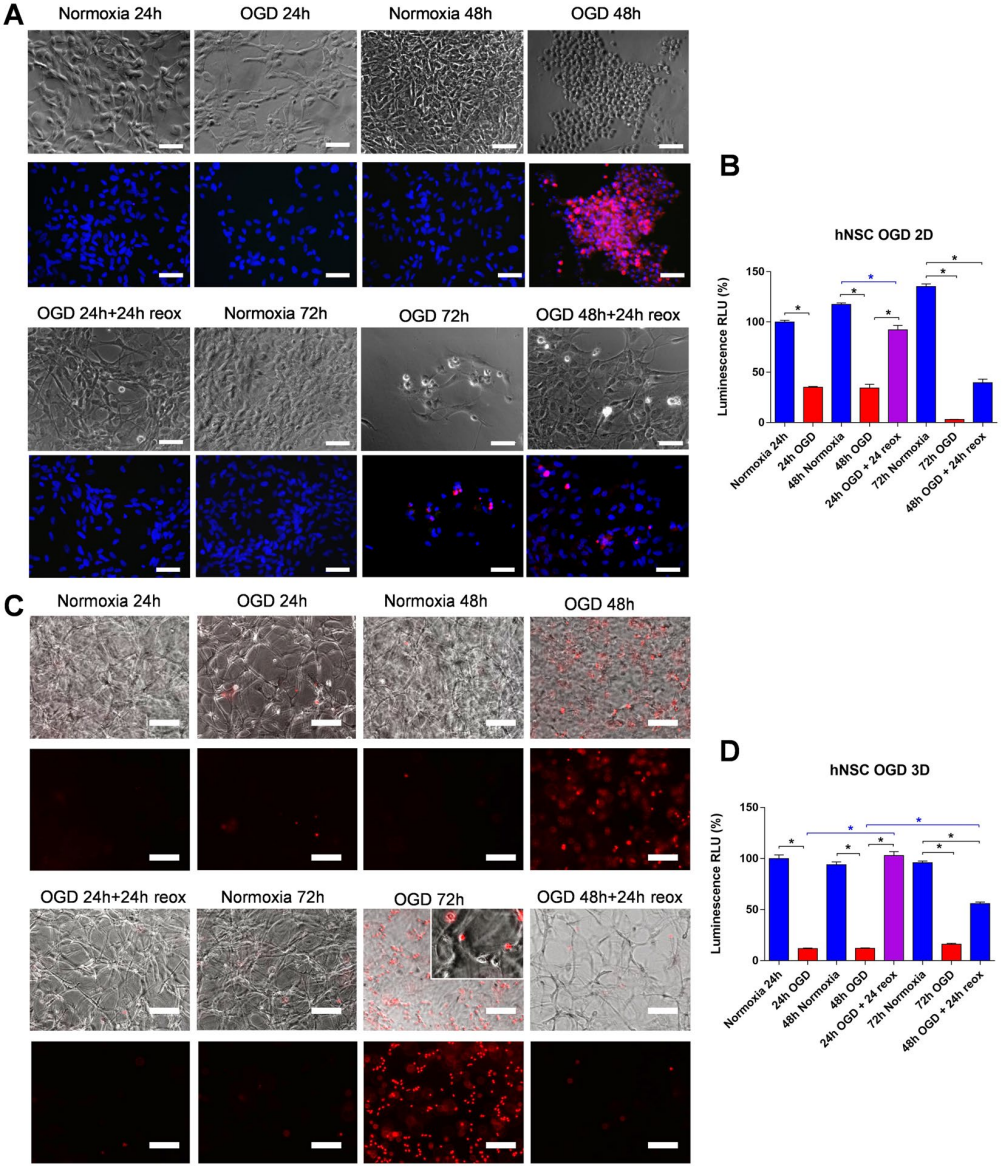
Effect of oxygen and glucose deprivation (OGD) and re-exposure to normal culture conditions to mimic hypoxia ischaemia and reperfusion in 2D and 3D hNSCs. (**A**) Effect of OGD on hNSCs morphology in 2D culture as observed by bright field microscopy and propidium iodide staining (red) under the indicated conditions. The inset shows presence of vacuoles in some cells after 24 hours OGD. Nuclei are in blue (Hoeschst staining). Scale bars are 100 µm. (**B)** hNSC viability quantified by ATP assay in cells grown in 2D following normoxia, OGD or reoxygenation (reox) at 24, 48 and 72 hours. (**C)** Effect of OGD on hNSCs morphology in 3D culture as observed by bright field microscopy and propidium iodide staining (red) under the indicated conditions as in (**B**). (**D)** hNSC viability quantified by ATP assay in cells grown in 3D under the indicated conditions as in (**B**). Note that significant recovery after 48 hours OGD is observed in 3D but not in 2D cultures. Data are expressed as mean ± S.E.M. of relative luminesce units (RLU, n = 6). (*Significance at p-value < 0.001). Scale bars are 100 µm.

hNSC survival in 2D and 3D culture was assessed after hypoxia and hypoglycaemia (OGD), followed by re-exposure to normal conditions to mimic hypoxia-ischaemia and reperfusion. To start with, we monitored the morphology of hNSCs in response to OGD in 2D cultures (Fig. 5A). Morphological changes indicative of cell stress started to be observed after 24 hour treatment, as indicated by the presence of vacuoles suggestive of autophagy, but occurrence of cell death as shown by propidium iodide staining was clearly visible at 48 hours (Fig. 5A). However, as indicated by the ATP assay, a strong decrease in hNSC metabolic activity (65% reduction) had occurred by 24 hours of OGD in 2D cultures (Fig. 5B). Interestingly, at least partial morphological recovery was observed when cells were re-oxygenated and re-exposed to standard culture medium after 24 h of OGD treatment, and this was reflected by a great increase in ATP levels. After 48 hours OGD, when most cells were dying, as indicated by their rounded morphology and extensive propidium iodide uptake, reoxygenation did not lead to an increase in ATP levels, but the surviving cells had largely reacquired a normal morphology (Fig. 5A,B). Only a few surviving cells were observed after 72 hours OGD in the 2D cultures, and, consistent with this, ATP levels were very low. In 3D cultures, ATP levels were also greatly reduced after OGD, but cell morphology was not greatly affected at 24 OGD and only a few propidium iodide-positive cells were observed (Fig. 5C,D). Much more extensive cell death was induced by 72 hours OGD, though some live cells extending processes were still present (Fig. 5C). In addition, in the 3D cultures hNSC recovery after re-oxygenation was observed even after 48 hours OGD, as indicated by the ATP assay (Fig. 5D).

**Figure 6:**
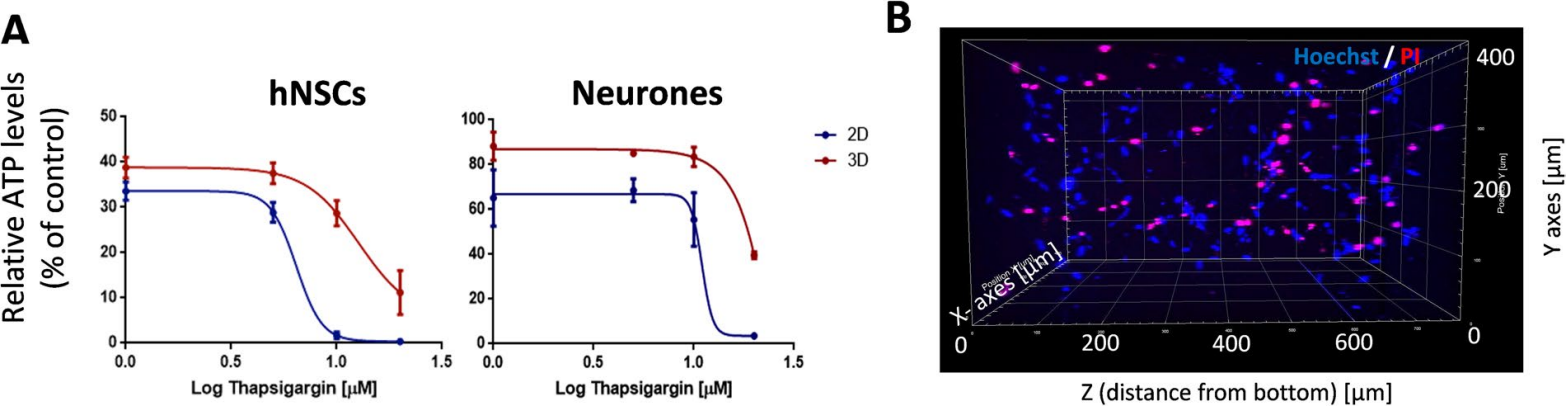
Dose-dependent effect of thapsigargin-induced calcium (Ca^2+^) dependent cell death in 2D and 3D hNSCs and neurones. (**A**) Dose response curves comparing the effect of thapsigargin treatment on viability of hNSCs and neurons in 2D (blue) and 3D (red) measured as percentage of ATP levels relative to untreated controls. Note differences in the thapsigargin concentration required to induce 50% cell death as compared to control wells, both in 2D and 3D. (**B)** hNSCs death across a 3D hydrogel following 5 μM thapsigargin treatment for 48 h assessed by live-stain with propidium iodide (PI, red); nuclei are in blue (Hoechst dye). Note that thapsigargin treatment causes cell death throughout the depth of hydrogel.

We then assessed the effect of increasing intracellular calcium on the metabolic activity of hNSCs and neurones in 2D and 3D using the ATP assay. Thapsigargin reduced hNSC metabolic activity and on the basis of the PI staining such reduction appeared to mirror an increase in cell death rather than a decrease in metabolic activity across the hNSC population (Fig. 6). Dose response experiments comparing the effect of thapsigargin on viability of undifferentiated and neuronally differentiated hNSCs in 2D and 3D showed higher resistance to thapsigargin-induced cell death in the 3D cultures (Fig. 6A). Furthermore, higher thapsigargin concentrations were required to induce 50% cell death in neurons than in undifferentiated hNSCs both in 2D and 3D. To rule out that the higher survival observed in 3D cultures might be due to limited diffusion of thapsigargin through the hydrogel, thapsigargin-treated 3D cultures were stained with propidium iodide to detect dying cells and imaged by confocal microscopy (Fig. 6B). This analysis revealed even distribution of dying cells throughout the depth of 3D hydrogels indicative of good drug diffusion within the hydrogel.

## Discussion

In order to study cellular and molecular processes that trigger and regulate injury to the human CNS, availability of *in vitro* cell models is crucial. Here we have developed hNSC 3-dimensional (3D) cultures that are suitable to model neural injury, further highlight the effect of dimensionality on cell behaviour and suggest that a 3D system could help to distinguish normal from cancerous cells. Importantly, this study shows difference in the responses of normal neural cells to two different types of injury in 3D and 2D, with the 3D system displaying lower susceptibility to damage. We suggest that by more closely mimicking tissue dimensionality these 3D models provide a better system to address both fundamental and applied research questions.

### hNSC morphology and gene expression are affected by the dimensionality of the culture

We have developed the hNSC 3D culture models following a biomimetic approach. Hydrogels derived from natural ECM polymers were utilized as preferable scaffolds as they are highly porous, their mechanical properties reflect the characteristics of CNS tissue, and they provide the necessary biological cues to which cells are exposed in their natural microenvironment^26^. Hence hNSCs can actively interact with the matrix and modify/remodel it. This is however a double-edged sword as hydrogels can be reorganized, shrunk and even completely degraded by the cells over the long time required for neuronal differentiation^27–29^. To circumvent this issue we developed an alternative strategy, where hNSCs were pre-differentiated in 2D and then incorporated into the hydrogel for final maturation. Seeding non-proliferative and nonmigratory cells in the hydrogel allowed it to maintain a stable 3D structure and to monitor live cell behaviour over a longer time. Furthermore, as hydrogels are transparent, cellular morphology and network formation can be imaged through the thickness of the hydrogel without sectioning it. This is a significant advantage as compared to the use of the rigid and non-transparent Alvetex scaffolds, where cells can be easily imaged without sectioning only at the surface of the scaffold. Although the rigidity of hydrogels and Alvetex is very different, a change in gene expression was observed in both systems within 5 days after seeding the hNSCs. As well as neural stem cell markers, some differentiation markers were significantly up-regulated in hNSCs seeded in hydrogels, as compared to 2D cultures, suggesting a shift in cell phenotype. In Alvetex the changes in gene expression at the same time after seeding were overall less marked, with a major difference as compared to hydrogel cultures being a reduction rather than an increase in *OLIG2*. Interestingly DCX-positive cells were still detected following neuronal differentiation in Alvetex, but not in hydrogel cultures. These results suggest that the hydrogel environment better supports a shift towards a more differentiated phenotype, and possibly an increased heterogeneity in hNSC 3D cultures.

### hNSCs and neuroblastoma cells respond differently to the 3D environment

hNSCs were not able to survive in pure collagen I hydrogels. This was in contrast not only to human neuroblastoma cells, but also to neural stem cells derived from other species that have been previously successfully grown in these conditions^22,30,31^ (chick, unpublished observation). The ability of the neuroblastoma lines studied here to thrive in collagen gels cannot be attributed to species differences. It is conceivable that differences in membrane protein expression may underlie different cell-matrix interactions that affect cell death/survival. This might be due either to the different embryonic origin of human neuroblastoma cells and hNSCs, neural crest- and neural tube-derived, respectively, or to the former being tumor cells. Pure Matrigel or mixed Matrigel/collagen I hydrogels, on the other hand, supported hNSCs growth, proliferation, differentiation and formation of complex neuronal networks. The mixed Matrigel/collagen hydrogels seemed to provide the best conditions for hNSC 3D culture; this is likely due to collagen I fibres that increase hydrogel stiffness and support for cell growth. However, at least 25% of laminin had to be present in the mixed gels, confirming hNSCs’ absolute requirement for this ECM component. NSCs express many of the laminin binding integrins, but not collagen I binding subunits, and in their niche are in contact with the basal lamina, which does not contain collagen I but is composed mainly of laminins, collagen IV, nidogen, and perlecan^32–35^. These proteins are abundantly present also in Matrigel. Lack of appropriate integrins could mediate cell death in the hNSCs grown in collagen I gels, as these molecules mediate not only adhesion, but also trigger many signalling pathways important for cell survival and proliferation^33,36,37^. Together, our study highlights the differences between human and animal NSCs that may to some extent explain failure in humans of pharmacological treatments effective in animal models of neural damage.

While hNSCs showed very strong dependence on laminin containing substrates, neuroblastoma cells were able to survive and actively proliferate in both pure collagen and Matrigel matrices forming aggregates with a tumour-like appearance. Given the importance of neuroblastoma interactions with ECM proteins^38^, the 3D cultures in hydrogels could better mimic the *in vivo* situation than the floating spheroid models widely used in cancer biology^39–41^. Furthermore, the hydrogel environment appeared to modulate expression of neural markers, CD133 and SOX2, which are also considered markers of “stemness” of tumour initiating cells, and are important prognostic markers of neuroblastoma progression and development^42–45^. Together, 3D cultures of undifferentiated neuroblastoma cells seem to be a more suitable model for cancer cell development than injury response studies. However, when neuronally differentiated, neuroblastoma cells could provide a convenient 3D human neuronal model for some applications (e.g. early compound screening), as they differentiate and mature within a shorter time frame (2 weeks) than hNSCs. Moreover neuroblastoma-derived neurones are more homogenous than hNSC-derived neurones, making data analysis and interpretation easier.

### 3D cultures can be used to model human neural damage

One of the basic requirements for establishing *in vitro* injury models is a fast and reliable method to evaluate and quantify cell viability/death. This is still challenging in 3D and the reliability of the assay can be affected by the type and size of the system. Here we have shown that the luminescence-based assay measuring ATP is suitable for monitoring hNSC metabolic activity in 3D cultures and that this is proportional to the number of cells present under normal culture conditions, indicating an effective penetration of the reagents throughout the hydrogel. However, in the OGD injury model, as more easily observable in the 2D cultures, changes in ATP levels cannot be taken to proportionally represent loss in cell viability, but rather reduced metabolic activity that can be reversed depending on the extent of damage, as suggested by the transient morphological changes. The reduction in hNSC metabolic activity with relatively limited cell death after 24 hour OGD *in vitro*, followed by significant recovery upon return to normal conditions, reflects the *in vivo* situation. It is indeed known that following hypoxic-ischaemic injury *in vivo* cells do not die immediately, and there is a window of opportunity for treatment before the onset of the more disruptive secondary damage^46,47^.

This study established that the timeframe for OGD treatment to induce injury to hNSCs *in vitro* both in 2D and 3D is 24–48 hours. The relatively long response time of hNSCs to OGD might be attributed to stem cell resistance to hypoxic-ischaemic injury in comparison with other progenitor cells and differentiated neurons^48,49^. It has been proposed that stem cells have a selective survival advantage by maintaining an undifferentiated state and low oxidative stress^50^, and that NSCs in their natural environment are exposed to mild hypoxic conditions^51^. However, another factor contributing to the slow response time to OGD might be the low solubility of oxygen in aqueous solutions, as well as slow equilibration of oxygen pressure between the gaseous and the aqueous phase of the system^52,53^, and this will need additional consideration to further optimize the model. Nonetheless, the current model provides a very promising tool for studying early responses to hypoxic-ischaemic injury at the cellular and molecular level as well as for testing putative neuroprotective pharmacological interventions for neural injury.

The most significant damage to the CNS following both traumatic injury and stroke is caused by secondary injury. One of the critical effectors of the progression of secondary injury is the increase in the levels of intracellular free Ca^2+^ that plays a critical role in triggering cell death^54–58^. This cell response to injury can be easily simulated in *in vitro* models by thapsigargin treatment. The results shown here suggest that both hNSC and neurones are more resistant to thapsigargin in 3D cultures. This is in line with previous studies in other cell types showing that cells respond differently to drug treatments in 3D as a result of their interactions with other cells and/or the ECM^59,60^. We ruled out that the difference observed in 3D response to thapsigargin may be due to poor penetration of the compound in the hydrogel. Indeed dead cells were present throughout the thickness of the culture, supporting the view that the lower sensitivity to thapsigargin in 3D cultures can be ascribed to differences in the culture cytoarchitecture rather than drug accessibility.

Together, the importance of the 3D human injury models we have established here is that they will provide a more complex and physiologically relevant system than currently available in 2D cultures, while being easier to set up and less variable than organoids for the study of human CNS damage at the cellular and molecular level and for evaluating putative neuroprotective drugs.

## Conclusions

We have shown that hNSCs do not thrive in collagen gel alone, a condition that supports rodent NSCs, and respond differently to the environment as compared to neurogenic neuroblastoma lines, with the former rapidly establishing networks, and the latter tumor-like aggregates. Therefore, allowing cells to establish interactions in multiple dimensions, rather than being constrained to interact largely in 2 dimensions as in monolayer cultures better reflects their *in vivo* behaviour. In 3D cultures, hNSCs rapidly change morphology and gene expression. These cultures allow diffusion of small molecules, as shown by accessibility of by propidium iodide and by the ability of thapsigargin to induce cell death throughout the hydrogel. Importantly, the 3D systems generated here appear to be suitable for modelling human neural injury, hence expanding the type and relevance of *in vitro* experiments that can be performed to study human CNS behaviour and decreasing the reliance of neuroscience research on animal models.

## Methods

### Reagents and cell incubation conditions

All cell culture reagents were obtained from Life Technologies unless stated otherwise. All cell lines and primary cultures were incubated at 37 °C in a humidified incubator with 5% CO_2_ unless stated otherwise.

### Human neuroblastoma cell lines

LAN-5 and IMR-32 cells were maintained in DMEM and SH-SY5Y in DMEM/F12 medium including GlutaMax, supplemented with 10% foetal bovine serum (FBS) and 1% penicilin/streptomycin (PS). Medium was changed every 2–3 days and cells were passaged upon reaching 70–90% confluency.

#### Neuronal differentiation of SH-SY5Y cells

For neuronal differentiation, SH-SY5Y cells were plated in standard culture medium (2 × 10^4^/cm^2^) onto plates pre-coated with diluted Matrigel (BD Biosciences; 1:30 in serum free medium) for 30 minutes at room temperature (RT). Differentiation was induced the following day by culturing in DMEM/F12 containing 1% PS and 10 μM retinoic acid (RA) for 5 days. Cultures were then maintained in DMEM/F12 with 1% PS and 50 ng/ml human recombinant BDNF for 5–10 days to allow neuronal maturation.

SH-SY5Y-derived 3D neuronal cultures were prepared by trypsinizing cells differentiated with RA for 5 days, pelleting and resuspending them to a final concentration of 6 × 10^5^ cells/ml of hydrogel prior to polymerisation (see below); 70 μl per well 96-well plates were used. After additional 2 days in the RA differentiation medium, cells were cultured for additional 9–16 days in the BDNF-containing medium as above. The medium was changed every 2 days.

### Human neural stem cells (hNSCs)

#### Isolation of hNSCs

All procedures involving human tissue were carried out in accordance with the Human Tissue Act 2006 with informed consent for study participation under ethical approval (NRES Committee London – Fulham, UK). The hNSCs from hindbrain from human embryos between 6–10 weeks old were collected through Human Developmental Biology Resource (HDBR, www.hdbr.org) tissue bank and isolated and grown as previously described^14,15^. The culture medium consisted of DMEM/F12 medium with GlutaMax (Life Technologies), 1% PS, 1% N2 supplement, 2% B27 supplement (both Life Technologies), 20 ng/ml human recombinant FGF2 (fibroblast growth factor 2), 20 ng/ml human recombinant EGF (epidermal growth factor; both Peprotech), 50 μg/ml bovine serum albumin (BSA) fraction V and 5 μg/ml heparin; laminin was added to the medium (10 μg/ml) as an alternative to coating the dishes.

#### Neuronal differentiation

A protocol previously modified from Sun *et al*.^14,16^ was used to induce neuronal differentiation of hNSCs^14,16^. After plating on Matrigel or laminin coated plates, hNSCs were maintained for 10 days in growth medium without EGF and then for further 7 days without EGF, FGF-2 and heparin to induce differentiation. Neuron maturation was then induced by culturing the cells in Neurobasal A medium supplemented with 1% PS, 1% L-Glutamine, 2% B27 and 10 ng/ml human recombinant βNGF with 10 ng/ml human recombinant BDNF (Peprotech) for 2–3 weeks.

### 3D cell cultures

#### Collagen I hydrogels

3D collagen hydrogel cultures were prepared as previously described^30^. Collagen type I (Col-I) solution (from rat tail tendons, ca 10 mg/ml in 0.2% acetic acid, BD Biosciences) was diluted with an equal volume of 2x PBS and then with cell culture medium to a final concentration of 2.7 mg/ml at 4 °C. After adjusting the pH to 7.4 with 1 N NaOH, the collagen hydrogel was mixed with the cell suspension in a 3:1 ratio to achieve 2 mg/ml final hydrogel concentration and 70 μl/well dispensed in 96-well plates. The hydrogel-containing cells were then allowed to polymerise at 37 °C for 30 minutes prior to adding 100 µl of culture medium.

#### Matrigel and Matrigel-collagen hydrogels

Cell suspensions in serum free medium were mixed with Matrigel (BD Biosciences) at 4 °C in a 1:1 ratio to obtain 3D Matrigel scaffolds with a final concentration of approximately 4 mg/ml Matrigel. Polymerisation was induced by incubation at 37 °C for 30 minutes as described above.

To prepare mixed Col-I/Matrigel cellularised hydrogels, a Col-I solution at pH to 7.4 was added to the Matrigel at 4 °C before mixing the hydrogel with the cell suspension and inducing polymerisation. Three final proportions of hydrogels were tested, with the following Matrigel/Col-1 concentrations (mg/ml): 3.4:1 mg/ml, 2.25:1.35 mg/ml, and 1.7:1.5 mg/ml. Following initial trials, Matrigel/Col-1 concentrations of 2.25:1.35 mg/ml were used unless otherwise specified.

#### 3D cultures in polystyrene scaffolds (Alvetex)

Tissue culture polystyrene scaffolds, Alvetex (diameter 15 mm, thickness 200 μm from Reinnervate, UK) were first hydrated in 70% ethanol for 1 min and then washed three times in sterile PBS. Prior to cell seeding, scaffolds were coated with laminin (10 μg/ml) diluted in serum-free medium for 1 h at room temperature. hNSCs were seeded at density 5 × 10^5^/scaffold in 50 μl of expansion medium and were allowed to attach for 1 h. After that the wells were topped up with medium, which was changed every 2–3 days. Samples were collected after 7 days in culture. For differentiation experiments cells were seeded on scaffolds as described above and cultured for 7 days to allow colonization of the full thickness of the scaffold, before the medium was changed to neuronal GF differentiation medium as described above.

#### hNSCs differentiation in 3D hydrogel cultures

To differentiate hNSCs towards neurons in 3D hydrogels, the cells were first plated in 2D and induced to differentiate as for the standard differentiation protocols as described above for 10 days. Primed cells were then detached enzymatically with Accutase, pelleted and gently resuspended in medium. They were then mixed with the hydrogel (Matrigel/collagen I mixture) at a final concentration of 1 × 10^6^/ml and the gel allowed to polymerise as described above. The medium was changed every 2–3 days and cells were kept in 3D cultures for 14 days before collection.

### Injury models

#### Calcium-dependent injury

Thapsigargin, a sarco/endoplasmic reticulum calcium ATPase (SERCA) inhibitor, was used to induce intracellular Ca^2+^ release in hNSCs and neurons. Cells were treated with thapsigargin (Sigma) dissolved in ethanol and diluted to final concentrations (up to 20 μM) in standard cell culture medium.

#### Oxygen/glucose deprivation (OGD) injury

To culture cells in hypoxic conditions, tissue culture well plates were placed in a semi-sealed incubation chamber with oxygen and carbon dioxide controllers (Biospherix) at 37 °C. Cells in cultures were exposed to hypoxic conditions with 0.5% O_2_ for 24–72 h. In the oxygen/glucose deprivation (OGD) model the cells were treated with no glucose medium (Life Technologies, without supplements) and hypoxia at the same time. In the reoxygenation model (reox) the cells were first treated like in the OGD group before they were returned to normoxia (21% O_2_ and 5% CO_2_) and normal growth medium (including glucose and supplements) for additional 24 hours to simulate reperfusion and secondary injury.

### Propidium iodide and Hoechst 33258 live staining

The live/dead staining was performed 48 hours after cell seeding as was described previously^61^. Hoechst 33258 (Sigma) dissolved in PBS and propidium iodide (PI) solution (Life Technologies) were added directly to cell culture medium at final concentrations of 2 μg/ml and 5 μg/ml respectively. Cells were incubated for 2 hours and then immediately examined with an inverted fluorescence microscope.

### BrdU staining

After 2 days in culture the medium was supplemented with 1 μM 5-bromo-2′-deoxyuridine (BrdU) for the last two hours of incubation. BrdU incorporation was stopped by removing the medium, cells were then washed with PBS and fixed with ice cold 70% ethanol for 20 min and incubated with 2 M HCl at 37 °C for 30 min to allow DNA denaturation. After neutralization with 0.1 M borate buffer (pH = 9) for 2–3 min and washing, the cells were treated with blocking solution made of 2% goat serum in PBS with 0.1% Triton X-100 for 1 h at room temperature. BrdU was detected with a monoclonal rat primary antibody (dilution 1:100, Serotec) diluted in blocking buffer and incubated with the sample overnight at 4 °C. Primary antibody was detected with a goat anti-rat secondary antibody conjugated with AlexaFluor594 (dilution 1:400, Life Technologies) diluted in blocking buffer and incubated 2 h at RT. For nuclei staining of all cells Hoechst 33258 (2 μg/ml) was added during the secondary antibody incubation.

### ATP assay

Cell viability in 3D hydrogel cultures was assessed by measuring the cell ATP content using the CellTiter-Glo3D® assay (Promega) according to the manufacturer’s protocol. Briefly, after adding equal volume of the 2x concentrated reagent to the culture medium, the samples were shaken vigorously for 5 min to aid the cell lysis process and then incubated at RT for 30 min. Luminescence was measured using the FLUOstar OPTIMA plate reader (BMG Labtech).

### Cell reduction potential assay

Cell viability assay measuring cell reduction potential by RealTime-Glo™ MT luminescence assay (Promega) was applied to assess cell viability over time in culture. The reagent was added to cell culture medium according to manufacturer’s protocol. Luminescence was measured repeatedly up to 48 hours after the addition of the reagent using the the FLUOstar OPTIMA plate reader.

### RNA analysis

Cells grown in monolayer or in Alvetex were washed with sterile PBS once before lysis with Trizol (Sigma) according to manufacturer’s protocol. Collagen hydrogel cultures were collected with a cell scraper and centrifuged at 7000 rpm for 5 min. Cell pellets were lysed with Trizol and triturated by pipetting until completely dissolved. Matrigel cultures were collected and incubated with Cell Recovery Solution (BD Biosciences) on ice for 1 hour with occasional rolling according to the manufacturer’s protocol to release the cells from the hydrogels. Mixed Matrigel/Col-1 hydrogel cultures were incubated with Accutase at 37 °C for 25 mins in order to release the cells from the hydrogels and washed twice with 1x PBS. The samples were then centrifuged at 6000 rpm for 5 min and pellets lysed with either Trizol or Qiagen buffer RLT. Total RNA was extracted using choloroform/phenol or Qiagen RNeasy mini spin column kits respectively. cDNA was prepared using MMLV reverse transcriptase (Promega) according to the manufacturers’ protocols.

The sequences of the primers used are listed in Supplementary Table 1. In reverse transcription-PCR experiments, all cDNAs were amplified for 35 cycles except for the house-keeping gene *GAPDH* (glyceraldehyde-3-phosphate dehydrogenase; 30 cycles) using the following conditions: 5 min 95 °C, 35 cycles of 30 s 95 °C, 30 s 52 °C–68 °C (depending on primers) and 30 s 72 °C, followed by 5 min 72 °C. PCR products were resolved on 1.5% agarose gel.

Real-time quantitative PCR (RT-qPCR) was performed with the ABI Prism 7500 sequence detection system (Applied Biosystems) using the QuantiTect SYBR Green PCR Kit (Qiagen) following the manufacturer’s instructions. The housekeeping genes *GAPDH* or 60S ribosomal subunit L19 (*RPL19*) were used as internal controls to normalize the expression levels. Data were analysed using the 2^−ΔΔCT^ method as described before^62^. No template controls, containing water instead of cDNA sample, and no reverse transcriptase controls were run with each RT-qPCR reaction and dissociation analysis was performed after each RT-qPCR reaction.

### Immunofluorescence

Briefly, cells grown in culture plates, on coverslips, or in 3D hydrogel cultures were fixed in 4% paraformaldehyde (PFA) solution in PBS, pH = 7.4 for 15 min (30 min for 3D cultures) at RT. After three rinses with PBS the cells were incubated with blocking solution composed of 10% FBS, 3% BSA in PBS with 0.2% TritonX-100 for 1 h at RT. Primary and secondary antibodies were diluted in blocking solution as specified in Supplementary Table 2. Incubation times were either 1 h at RT or overnight at 4 °C for primary, and 1 h at RT for secondary antibodies. Hoechst 33258 (2 μg/ml) was added during the secondary antibody incubation to counterstain cell nuclei. After the final three washes in PBS the coverslips were mounted with an aqueous based mounting medium (Hydromount, National Diagnostics). PFA-fixed 3D polystyrene scaffolds were cryosectioned and immunostained following a similar protocol. Images were acquired using an inverted microscope Olympus IX71 equipped with monochrome ORCA-R2 digital camera (Hamamatsu Corp.). Confocal laser scanning microscopy was performed using the Zeiss, LSM 710 confocal system (Carl Zeiss). All image processing and analysis was performed in Fiji/ImageJ software^63^ or Imaris software for 3D image reconstruction. In the case of 3D imaging, due to the thickness of the hydrogel (~800 µM), fluorescent signal gradually weakens with depth, hence excitation laser strength was optimised to image throughout gels.

### Statistical analysis

Each experiment was performed in biological triplicates unless stated otherwise. Statistical analysis was carried out with one-way ANOVA followed by multiple comparison Tukey’s test or unpaired Student’s t-test. GraphPad Prism 7 (GraphPad software Inc. La Jolla, CA, USA) was used for all statistical calculations. The results are expressed as mean ± standard error of the mean (S.E.M). Differences were considered to be significant if p < 0.05.

## Supplementary information

Supplementary Tables and Figures.
