# Supplementary material for: Modelling human CNS injury with human neural stem cells in 2- and 3-Dimensional cultures
Source: Sci Rep. 2020 Apr 22;10:6785. doi: 10.1038/s41598-020-62906-y (PMC7176653; doi:10.1038/s41598-020-62906-y)
Supplement: Supplementary file 1 — Supplementary Tables and Figures. [file 41598_2020_62906_MOESM1_ESM.pdf]

Supplementary Tables and Figures

Modelling human CNS injury with human neural stem cells in 2- and 3-Dimensional cultures

Barbora Vagaska^, Olivia Gillham^, and Patrizia Ferretti*

Stem Cells and Regenerative Medicine Section, UCL Great Ormond Street Institute of Child Health, University College London, WC1N 1EH, UK

^These authors contributed equally to the study

Running title: Modelling human neural damage: 2D versus 3D

Corresponding author: *Patrizia Ferretti, Stem Cells and Regenerative Medicine Section, UCL Institute of Child Health, 30 Guilford Street, London WC1N 1EH, UK

Tel: (+44) 020-7905 2372 (direct line) Fax: (+44) 020 7905 2953 E-mail: p.ferretti@ucl.ac.uk

Supplementary Table 1. List of primer sequences.

Name<br>Accession No.		Sequence
**GAPDH**<br>NM_001289746	**Fw**<br>**Rv**	CCTTCATTGACCTCAACTACATGGT<br>CTAAGCAGTTGGTGGTGCAGGA
**ACTB (b-actin)**<br>NM_001101	**Fw**<br>**Rv**	GAAGGTAGTTTCGTGGATGC<br>CCCTGGAGAAGAGCTACGA
**RPL19 (L-19)**<br>NM_000981.3	**Fw**<br>**Rv**	GCGGAAGGGTACAGCCAAT<br>CAGGCTGTGATACATGTGGCG
**PROM1 (CD133)**<br>NM_001145852	**Fw**<br>**Rv**	CAGAGTACAACGCCAAACCA<br>AAATCACGATGAGGGTCAGC
**SOX2**<br>NM_003106	**Fw**<br>**Rv**	GCCGAGTGGAAACTTTTGTCG<br>GCAGCGTGTACTTATCCTTCTT
**NES (NESTIN)**<br>NM_006617	**Fw**<br>**Rv**	CAGCGTTGGAACAGAGGTTGG<br>TGGCACAGGTGTCTCAAGGGTAG
**PAX6**<br>NM_001258465	**Fw**<br>**Rv**	GGGCAATCGGTGGTAGTAAA<br>CTAGCCAGGTTGCGAAGAAC
**SLC1A3 (GLAST)**<br>NM_001289940	**Fw**<br>**Rv**	CTCACAGTCACCGCTGTCAT<br>CCATCTTCCCTGATGCCTTA
**BLBP (FABP7)**<br>NM_001446	**Fw**<br>**Rv**	CCAGCTGGGAGAAGAGTTTG<br>CTCATAGTGGCGAACAGCAA
**OLIG2**<br>NM_005806	**Fw**<br>**Rv**	CAGAAGCGCTGATGGTCATA<br>TCGGCAGTTTTGGGTTATTC
**GFAP**<br>NM_001242376	**Fw**<br>**Rv**	GAAGCTCCAGGATGAAACCA<br>ACCTCCTCCTCGTGGATCTT
**TUBB3 (β3-TUBULIN)**<br>NM_001197181	**Fw**<br>**Rv**	CTCAGGGGCCTTTGGACATC<br>CAGGCAGTCGCAGTTTTCAC
**NEFH (NF-H)**<br>NM_021076	**Fw**<br>**Rv**	ACGCCCTGAAGTGCGACGTG<br>CTCCAGCTCAGAGCGCTGCC
**ENO2 (NSE)**<br>NM_001975	**Fw**<br>**Rv**	CTGATGCTGGAGTTGGATGG<br>CCATTGATCACGTTGAAGGC
**KCND3 (Kv4.3, potassium channel)**<br>NM_004980	**Fw**<br>**Rv**	GCCTCCGAACTAGGCTTTCT<br>CCCTGCGTTTATCAGCTCT
**MAP2**<br>NM_002374	**Fw**<br>**Rv**	CCACCTGAGATTAAGGATCA<br>GGCTTACTTTGCTTCTCTGA
**GAD1**<br>NM_000817.2	**Fw**<br>**Rv**	GTCGAGGACTCTGGACAGTA<br>GGAAGCAGATCTCTAGCAAA
**SLC6A4**<br>NM_001045.5	**Fw**<br>**Rv**	GCCTTTTACATTGCTTCCTA<br>CCAATTGGGTTTCAAGTAGA
**CHAT**<br>NM_001142933.1	**Fw**<br>**Rv**	ACTGGGTGTCTGAGTACTGG<br>TTGGAAGCCATTTTGACTAT
**TH**<br>NM_199293.2	**Fw**<br>**Rv**	TCATCACCTGGTCACCAAGTT<br>GGTCGCCGTGCCTGTACT

Supplementary Table 2. List of primary and secondary antibodies used for immunostaining**.**

Primary Antibodies – Target antigen	Host	Company	Cat. Number	Dilution
**GFAP (Glial Fibrillary Acidic Protein)**	rabbit	Millipore	AB1540	1/400
**NESTIN**	rabbit	Millipore	ABD69	1/500
**NESTIN**	mouse	Abcam	ab22035	1/500
**SOX2**	rabbit	Millipore	AB5603	1/200
**ß3-TUBULIN**	mouse	Promega	G712A	1/500
**Vimentin**	mouse	Dako	M0725	1/500
**Doublecortin**	rabbit	Invitrogen	48-1200	1/200
**MAP2**	mouse	Life Technologies	13-1500	1/200
**NeuN**	mouse	Millipore	MAB377	1/100
**NF200**	rabbit	Sigma	N4142	1/100
**Synaptic vesicle glycoprotein 2A (SV2)**	mouse	DSHB	SV2	1/50
**BrdU**	rat	Serotec	OBT0030	1/400
**Phalloidin +Alexa488 (actin binding)**	N/A	Invitrogen	A12379	5U/100µl

Secondary antibodies- target species	Host	Fluro chrome	Company	Cat. Number	Dilution
**anti-mouse IgG**	goat	Alexa594	Molecular Probes	A-11020	1:400
**anti mouse IgG**	donkey	Alexa488	Molecular Probes	A-21202	1:400
**anti rabbit Ig**	donkey	Alexa488	Molecular Probes	A-21206	1:400
**anti rabbit Ig**	donkey	Alexa568	Molecular Probes	A-110042	1:400
**anti rat Ig**	goat	Alexa 594	Molecular Probes	A-11007	1:400

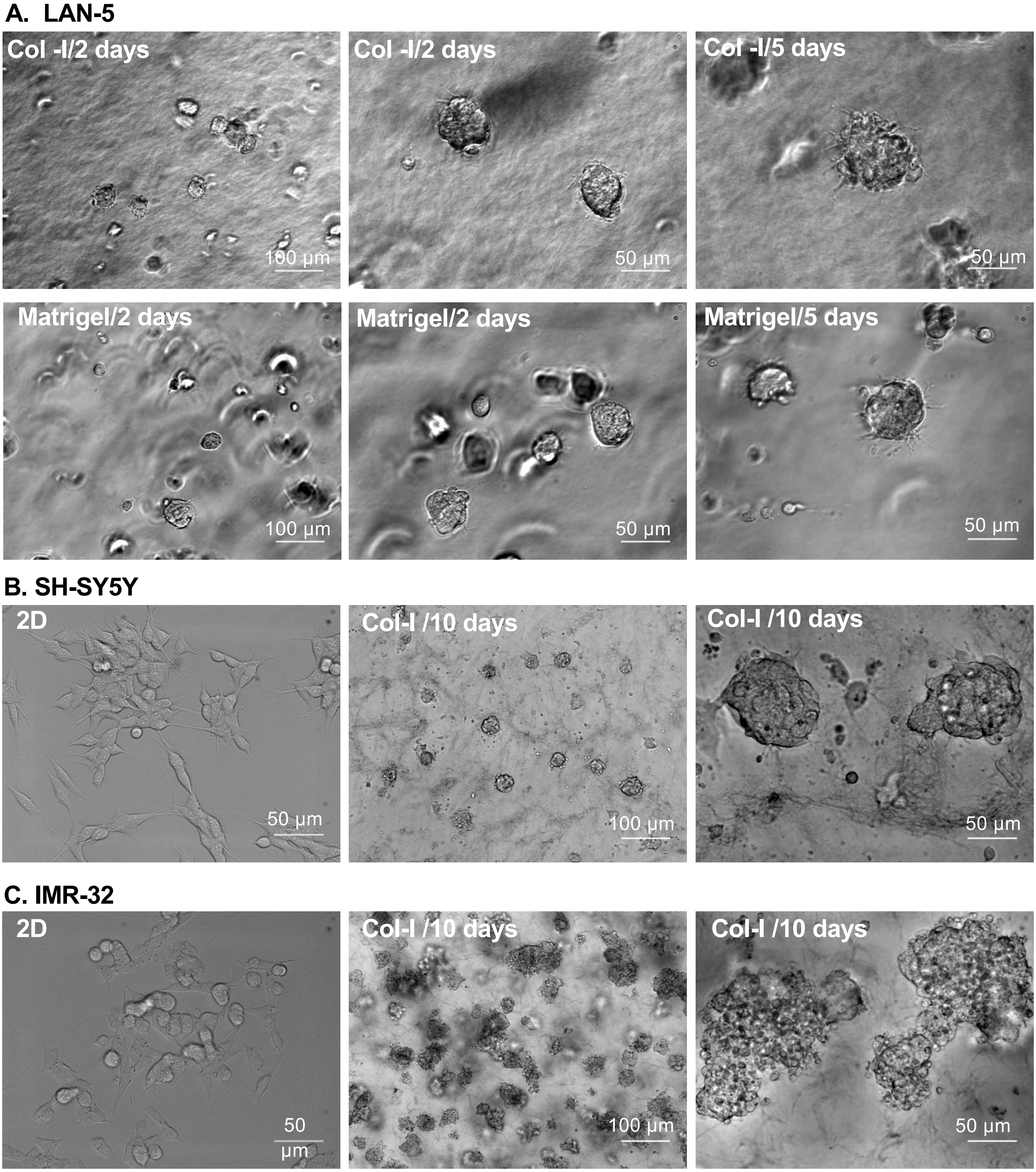

Supplementary Figure 1. Neuroblastoma cells in 3D cultures. Representative bright field images of 3 human neuroblastoma lines. **A)** LAN-5 cells grown in 3D Matrigel or Col-I (Collagen 1) hydrogels for 2 and 5 days show morphological changes and formation of tumor-like aggregates. **B-C)** Similar changes in morphology are observed also in SH-SY5Y (B) and IMR-32 (C) neuroblastoma lines grown in 3D Col-I for 10 days. Note that cells remain spread when grown in 2D.

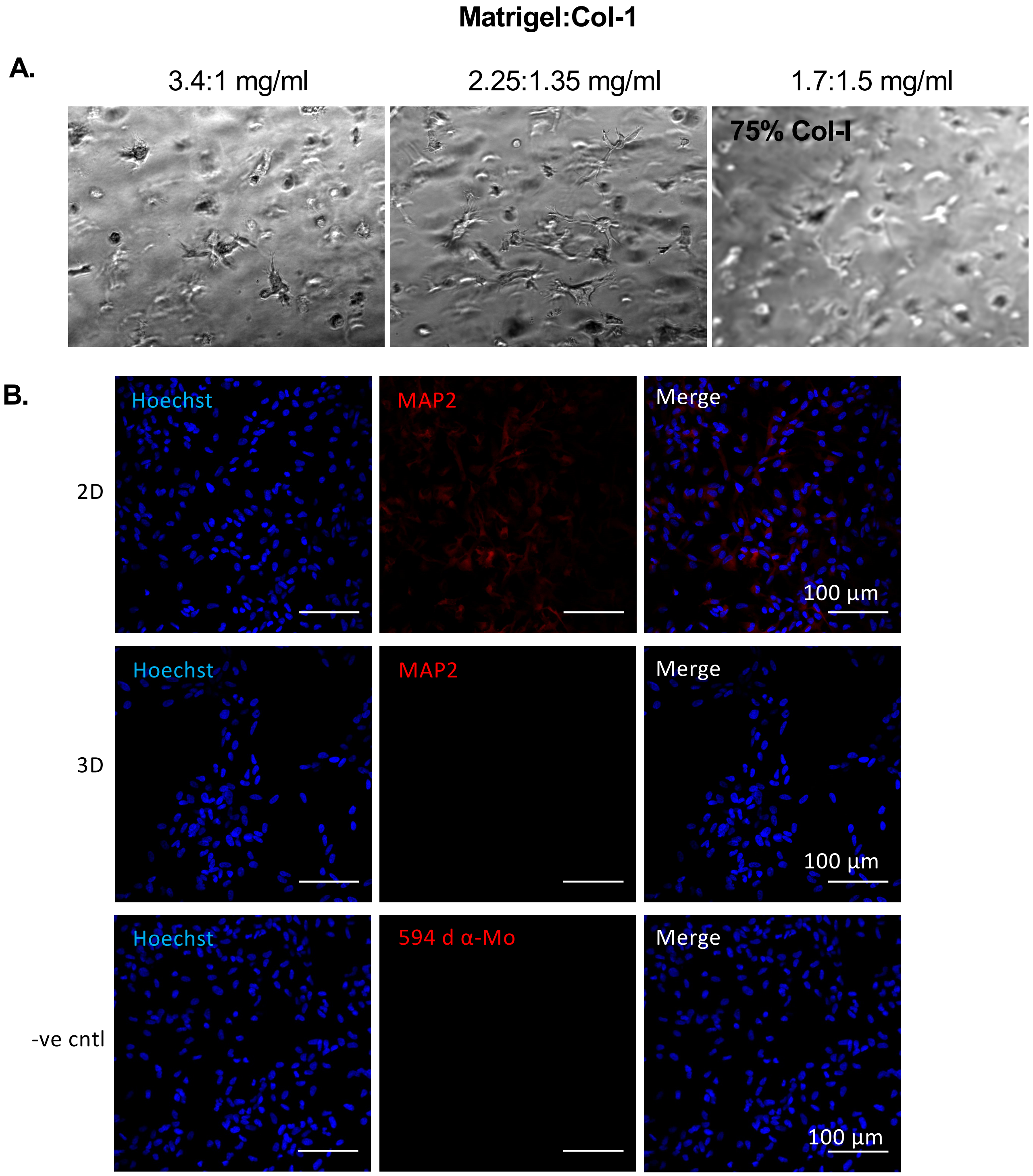

Supplementary Figure 2. Live imaging and immunostaining of hNSCs in 3D cultures. A) Representative live bright field images of cells seeded in Collagen-I (Col-I) / Matrigel hydrogel containing different amounts of Col-I after 5 days in culture. **B)** Expression of MAP2 expression in hNSCs in 2D and 3D hydrogel (Matrigel/Col-I, 2.25/1.35 mg/ml) as assessed by immunostaining 5 days after seeding. No staining is observed when the primary antibody is omitted (-ve cntl). Nuclei (blue) are detected by Hoechst dye staining.

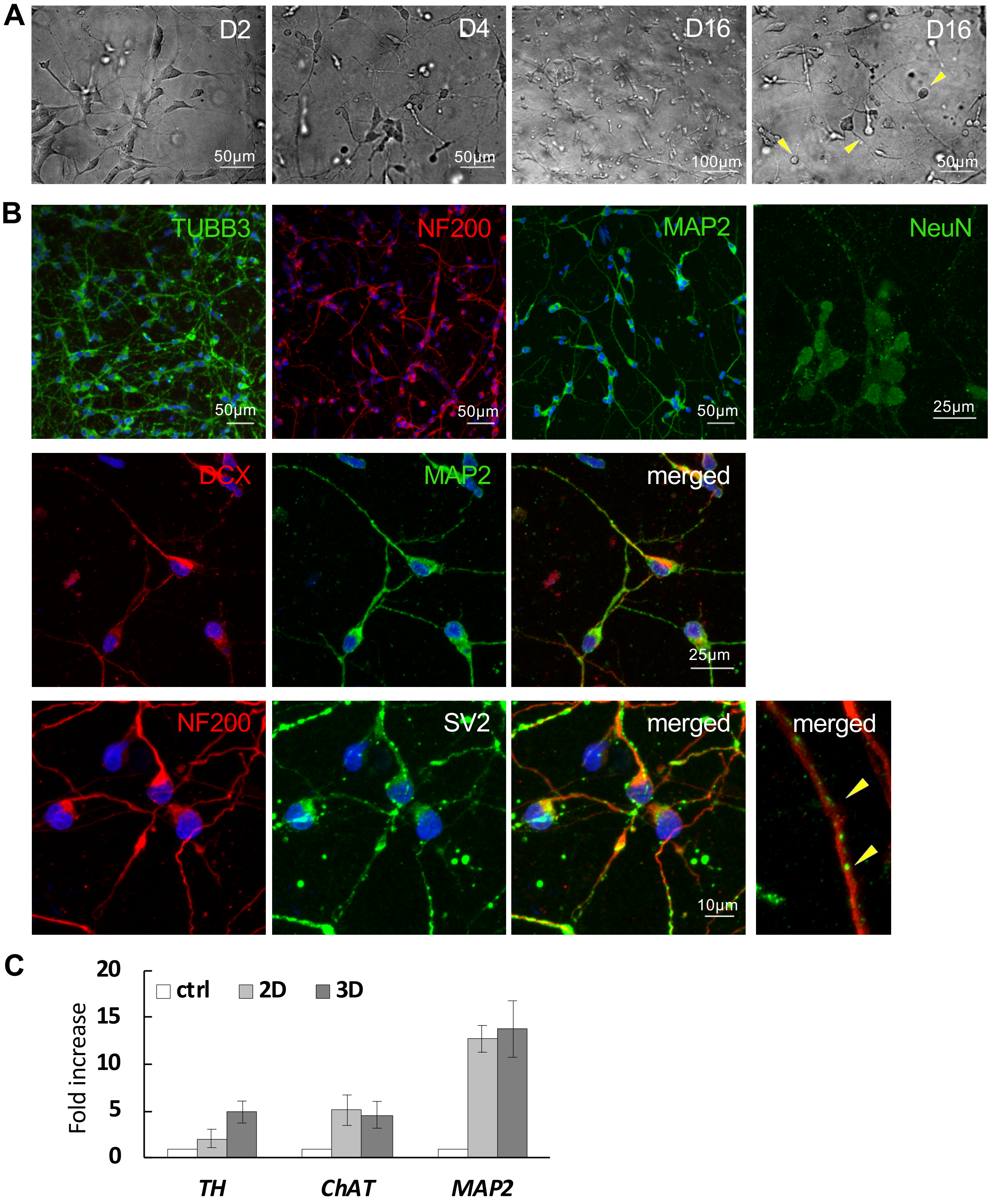

Supplementary 3. Neuronal differentiation of neuroblastoma cells in 3D cultures. (A) Bright field images show morphological changes during SH-SY5Y cells after 2, 4 and 16 days of neuronal differentiation in 3D cultures following 5 days retinoic acid in 2D cultures. Yellow arrowheads indicate cells with unipolar morphology. **B)** Immunocytochemical assessment of neuronal markers expression in SH-SY5Y cells differentiated in 3D gels for 14 days: ß3-TUBULIN (TUBB3), neurofilaments (NF200), MAP2, NeuN and doublecortin (DCX). Synapse specific protein (SV2) staining shows presence of synaptic structures and functional vesicle transport (yellow arrows). Nuclei are counterstained in blue with Hoechst 33258. **C)** Expression of tyrosine hydroxylase (*TH*), choline acetyl transferase (*CHAT*) and *MAP2* as markers of neuronal subtypes and differentiation after 14 days in culture (5 days retinoic acid in 2D plus 9 days of neuronal differentiation in 3D) was assessed by RT-qPCR. The y-axis indicates fold changes in mRNA expression in relation to control undifferentiated SH-SY5Y in 2D cultures (ctrl). Data expressed as mean ±SEM, n=3 (biological replicates). Significant increase ($p<0.01$) of neuronal markers is induced upon differentiation in both 2D and 3D, with the exception of TH that in 2D is significantly different from 3D but not from controls.

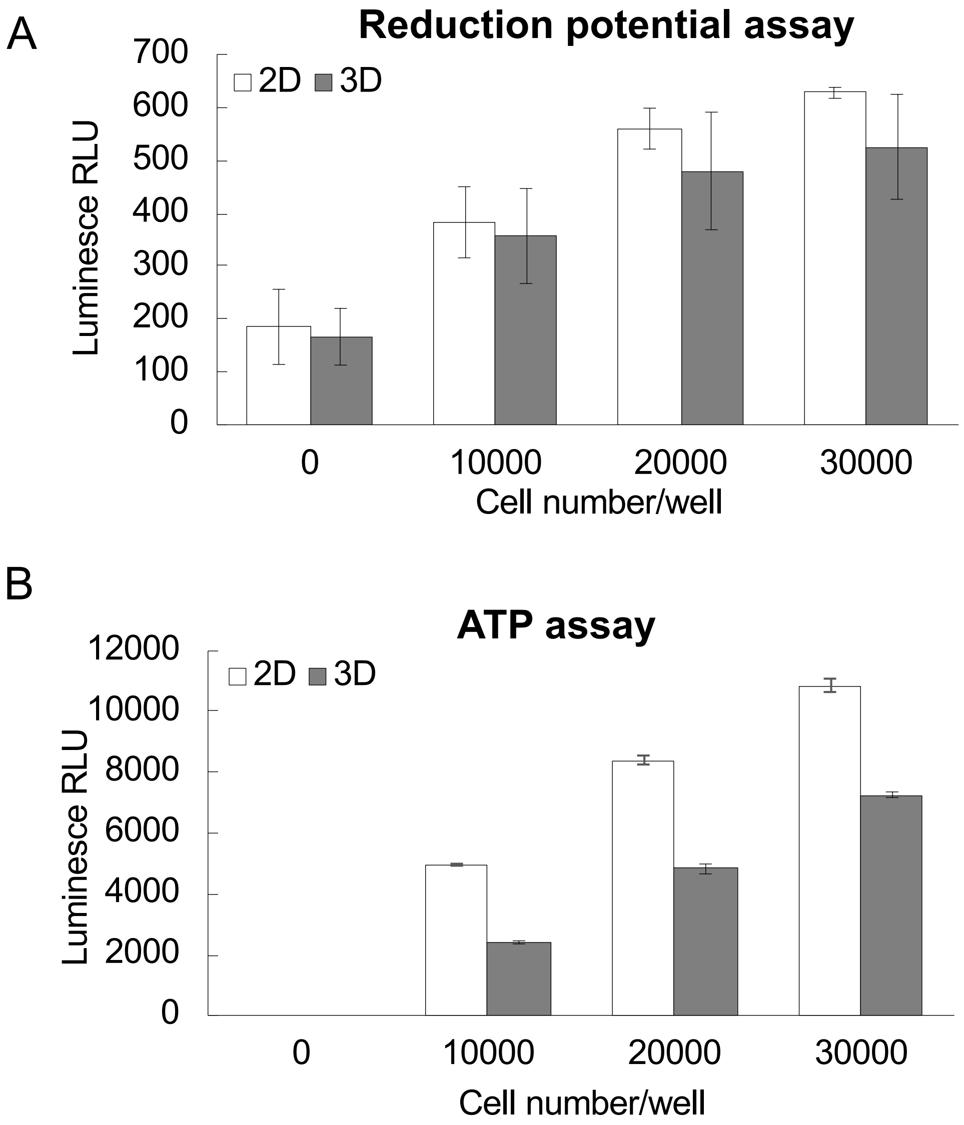

Supplementary Figure 4. Measurement of cell viability in 3D cultures with luminescence assays. Comparison of **(A)** reduction potential and **(B)** ATP content in 2D and 3D hNSCs cultures by luminescence assays to monitor cell viability. Data are expressed as mean ± S.E.M. of relative luminescence units (RLU, n=3).
